# Supplementary figures and images for: Charge and hydrophobicity are key features in sequence-trained machine learning models for predicting the biophysical properties of clinical-stage antibodies
Source: PeerJ. 2019 Dec 18;7:e8199. doi: 10.7717/peerj.8199 (PMC6967001; doi:10.7717/peerj.8199)

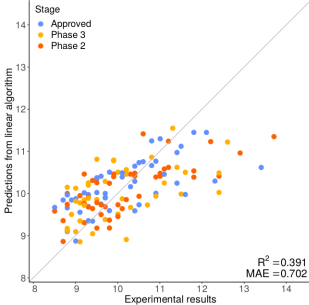

(a) HIC

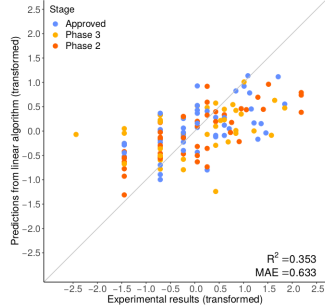

(b) SMAC

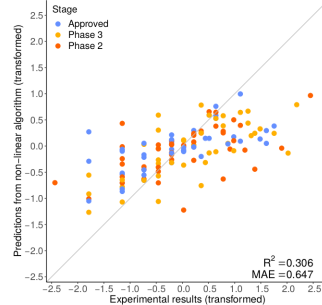

(c) CIC

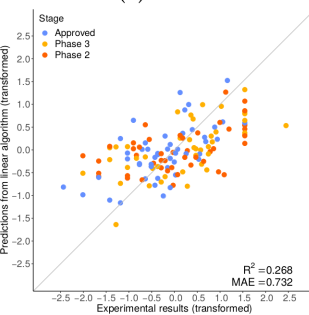

(d) ACSINS

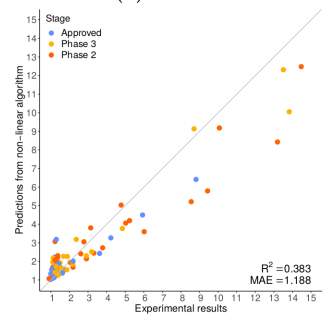

(e) ELISA

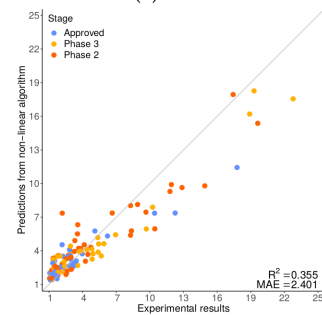

(f) BVP

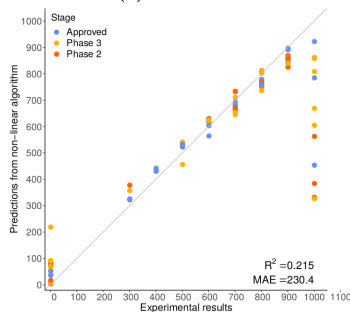

(g) SGAC-SINS

Supplement: Supplemental Information 1 — The scatter graphs demonstrate the predictive power of each model, where the original experimental value is on the x axis, and the prediction is on the y axis. The closer each data point is to y = x the better the prediction. For example, in Figure A, the predictions for HIC are generally close to the y = x line until x = 11 min. Above this point the model begins to lose predictive power, and the values begin to fall away from the diagonal. Where applicable, both the x and y axis are reported in terms of the mathematically transformed values. [file peerj-07-8199-s001.pdf]

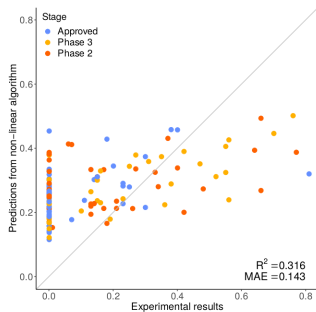

(a) PSR

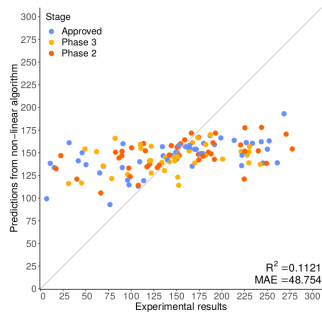

(b) HEK

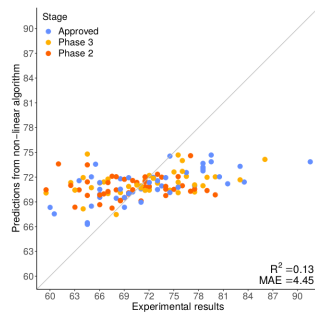

(c) DSF

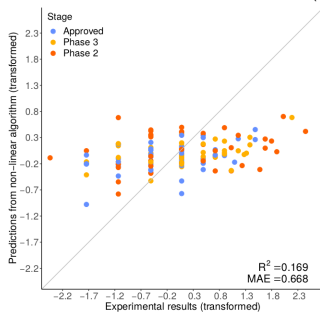

(d) CSI

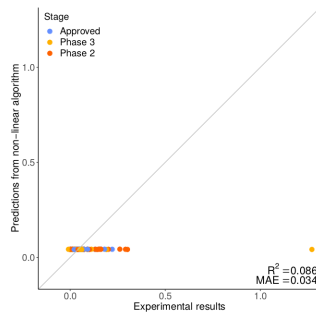

(e) ACC STAB

Supplement: Supplemental Information 2 — As in Figure S1, the scatter graphs demonstrate the predictive power of the models. In comparison, these models do not perform as well and therefore have lower predictive power. [file peerj-07-8199-s002.pdf]

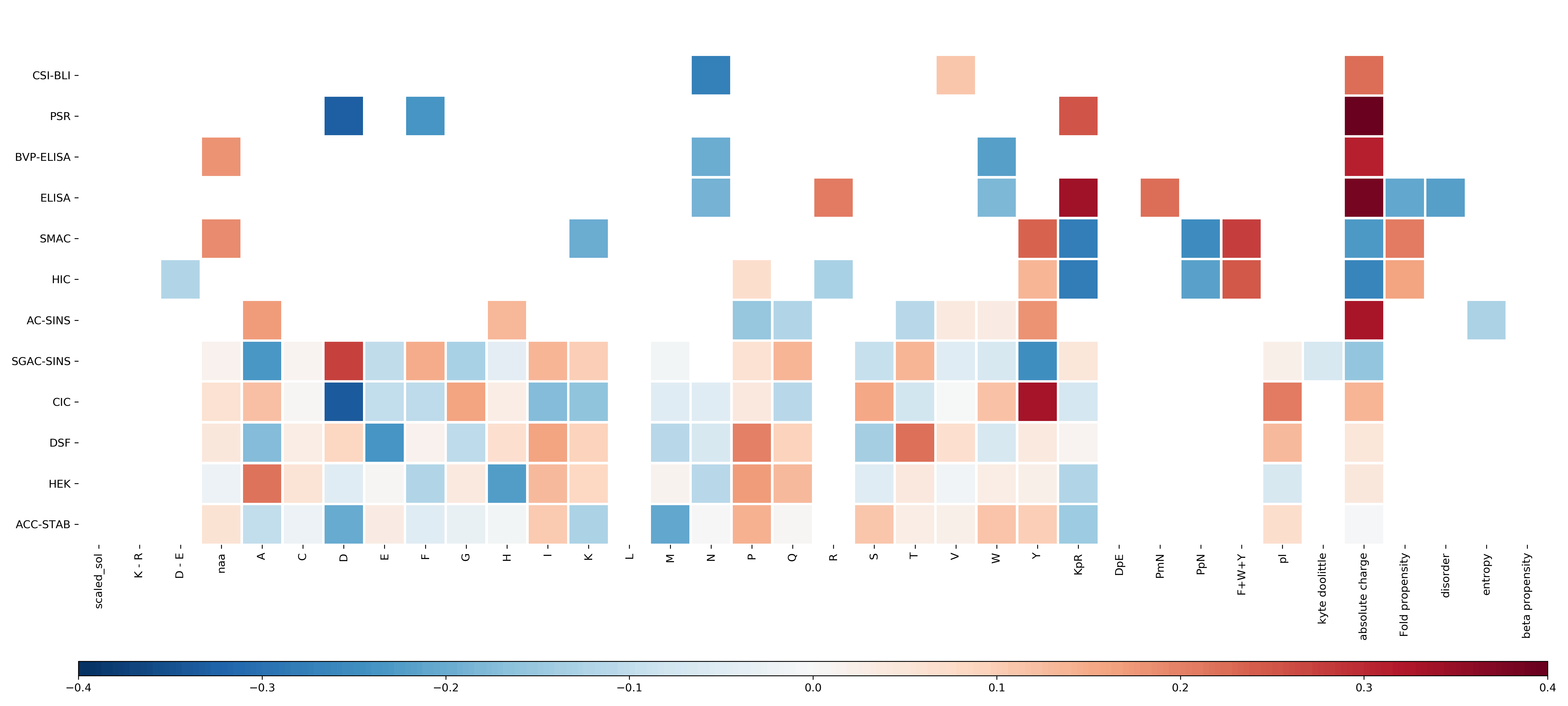

Supplement: Supplemental Information 3 — In comparison, only features selected for by each algorithm are chosen to elucidate the relationship between the features selected, and the correlation coefficient. Of note, features associated with charge and hydrophobicity are prominent both for their generally high correlation values, but also the regularity at which they are chosen by each algorithm. Dark red values indicate a stronger positive correlation, and dark blue values indicate a stronger negative correlation. [file peerj-07-8199-s003.png]

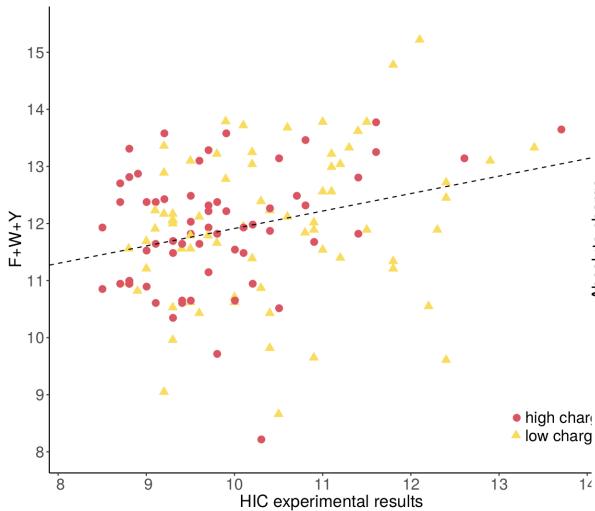

(a) Aromatic content

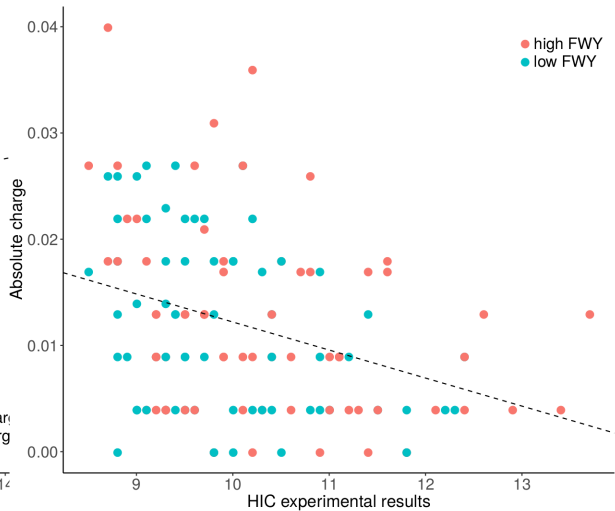

(b) Charge

Supplement: Supplemental Information 4 — In (A), the aromatic content—here defined as the combined composition value for the amino acids F, W and Y - shows a general positive correlation with the HIC experimental score for that sequence. Sequences with an above average absolute charge value, in the context of the mAb137 dataset, are coloured red, and those with less yellow. In (B), there is a general negative correlation between the absolute charge—calculated from the number of charged residues D, E, K and R within the sequence—and HIC experimental score. Sequences with an above average aromatic content are coloured pink, and those with a below average aromatic content are coloured blue. [file peerj-07-8199-s004.pdf]
